# Acute Urinary Retention in the First-trimester of Pregnancy: A Case Report

**DOI:** 10.7759/cureus.23057

**Published:** 2022-03-11

**Authors:** Chenyang Dai, Jiali Peng, Rufang Chen

**Affiliations:** 1 Obstetrics and Gynecology Department, The First People's Hospital of Foshan, Foshan, CHN

**Keywords:** knee-chest prone position, catheterization, retroverted uterus, pregnancy, acute urinary retention

## Abstract

Acute urinary retention (AUR) is a rare occurrence during pregnancy. If not dealt with in time, it can lead to bladder rupture, miscarriage, or even uterine rupture and other serious consequences that endanger the health of both mother and fetus. Many risk factors have been identified. To better understand the etiology and treatment of urinary retention during pregnancy, we report on one pregnant woman with AUR who presented at 11+1 weeks of gestation due to uterus retroversion.

## Introduction

Acute urinary retention (AUR) is defined as a sudden and painful inability to voluntarily void urine [1]. AUR is a rare but serious complication of pregnancy. It can lead to serious consequences, such as acute renal failure, spontaneous abortion, permanent bladder dysfunction, and bladder rupture [2], and occurs more often between 10 and 16 weeks of gestation [3,4]. According to previous reports, AUR occurs in approximately 1 in 3,000 pregnant women and is often associated with uterine incarceration [5]. However, as shown in our report, even without uterine entrapment, retroversion of the uterus itself can cause AUR.

## Case presentation

**Figure 1:**
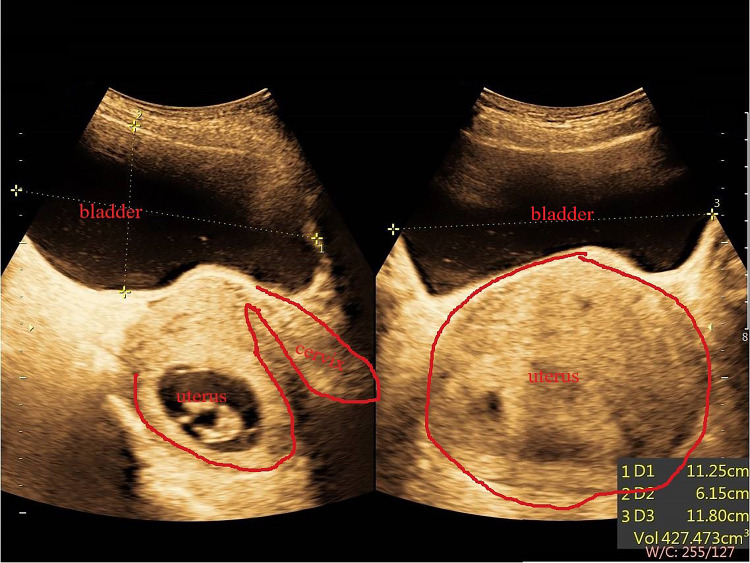
Abdominal ultrasound shows a retroverted uterus and a full bladder. Abdominal ultrasound shows a retroverted uterus and a full bladder. The left image is the longitudinal section of the probe, and the right image is the transverse section of the probe.

A 32-year-old primigravida was admitted to the Obstetrics Department with AUR at 11+1 weeks of gestation on December 27, 2021. The patient’s pregnancy had been normal until six days ago when she experienced urgency, frequent urination, and nocturia at least 10 times per night, associated with a sensation of incomplete bladder emptying and dysuria. The illness, personal, and family history of this patient were unremarkable. Physical examination showed that a dramatically distended bladder was palpable above the pubic symphysis, and gynecological examination detected fullness in the posterior fornix and anterior displacement of the cervix. On bimanual examination, the retroverted uterus impacted in the pelvis was found. Routine urinalysis and assessment of kidney function (December 27, 2021) were negative. The ultrasound (December 27, 2021) detected a retroverted uterus. A gestational sac was seen in the uterine cavity, and a fetal echo was seen in the sac. The fetal heartbeat is visible. CRL is about 43 mm, and NT is 1.5 mm. The echoes of the uterine wall were uniform, the bilateral ovaries were visible, and no obvious abnormal mass echo was found in the bilateral appendages. The lower rod of the bladder is trapped by the pregnant uterus and contains approximately 427 cm^3^ urine (Figure 1).

**Figure 2:**
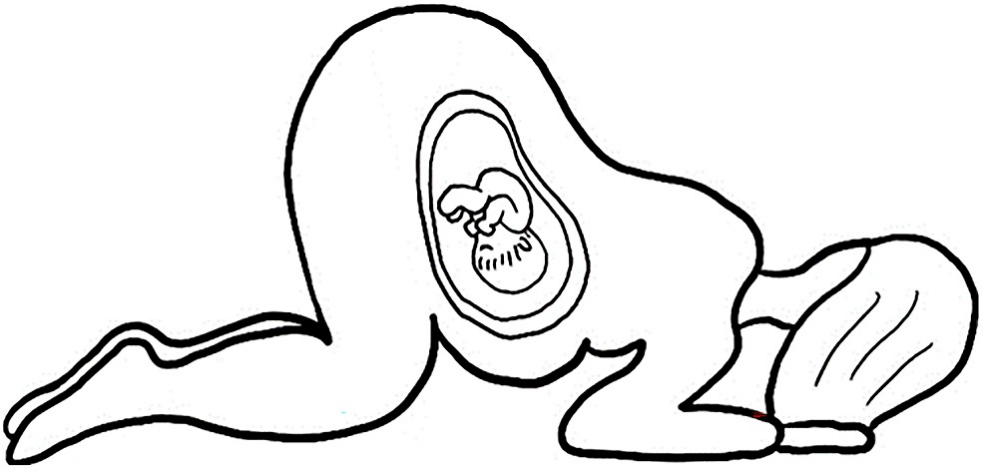
Knee-chest prone position.

The patient was diagnosed with AUR during pregnancy. The patient experienced rapid relief from her bladder discomfort after being inserted with a urinary catheter, with prompt drainage of 450 ml of clear urine. The catheter was continued indwelled and clipped intermittently five days later to exercise bladder function. Meanwhile, the patient was guided to adopt the knee-chest prone position for 15 min, 3 times a day to improve uterine retroversion (Figure 2). Ultrasound showed no pelvic mass, and the uterine polarity changed significantly seven days later. The patient's symptoms improved significantly, and she was able to void normally and was discharged from the hospital after the indwelling catheter was removed seven days later. The patient was advised to adopt a knee-chest prone position and limit fluid intake before sleep, as before, to prevent an AUR relapse during the next pregnancy. Thereafter, the patient has a regular obstetrics examination in our hospital. Her symptoms did not recur again, and her pregnancy remained uneventful until now.

## Discussion

The etiology of AUR can be broadly classified into infectious, pharmacological, neurological, anatomical, myopathic, and functional categories [6]. In addition, as shown by the findings of Chen et al., maternal age >35 years, preterm birth, and first delivery were significantly associated with AUR [7]. While the most studied pathogenesis of AUR in pregnancy is anatomy, this includes endometriosis, pelvic adhesions, congenital malformations of the uterus, fibroids, or anatomical variations in the shape of the pelvis [4]. And interestingly, most cases of AUR are noted to take place in the middle of the night [8]. In the present case, the patient is a primipara, but no other high-risk factors have been found. The proposed mechanism is that, under the action of hormones, the glands in the cervical canal become hyperplasia and hypertrophy, which makes the cervix gradually soften after pregnancy, increasing the chance of uterine retroversion. Then the cervix presses against the lower part of the bladder and interferes with urethral drainage without causing deformation or compression of the urethra. This suggests that even without confinement, uterine inversion during pregnancy can cause AUR.

AUR can have a variety of clinical manifestations. According to the available data, the most common symptoms of AUR during pregnancy are urinary incontinence, incomplete urination, vaginal bleeding, miscarriage, painful urination, rectal compression, and constipation [4,9]. However, rare symptoms such as lower extremity swelling, severe hypertension, hydronephrosis, and asymptomatic AUR are easily overlooked during consultation [2,10]. Physical examination findings mainly include a low fundal height for the age of gestation, bladder distension, and palpation of a mass that can induce urgency. It is often difficult to see the cervix on a vaginal exam because it is hidden behind the symphysis pubis [4], as also shown in our case.

There is no simple diagnostic test to determine the cause of urinary retention during pregnancy. Apart from clinical manifestations and physical examination, auxiliary examinations such as ultrasound, magnetic resonance imaging (MRI), and endoscopy are useful tools for the diagnosis of a retroverted gravid uterus and AUR, and the use of sonography as a non-invasive diagnostic tool should be encouraged in a larger group of patients first [11]. In this study, typical signs and symptoms of the genitourinary system helped us to make a preliminary judgment, and an ultrasound image provided final confirmation. MRI can be used in complex cases and as a more accurate test to rule out other possible diagnoses. As Dierickx et al. [12] suggested, MRI was the imaging modality of choice for uterine incarceration, and MRI is recommended prior to manual or colonoscopy-assisted reduction to rule out anterior incarceration. Using cystoscopy as a diagnostic tool, it is recommended to exclude the presence of urinary vegetations, as the cause of AUR in pregnancy is external pressure, and cystoscopy is not very meaningful.

Bladder decompression by insertion of an indwelling Foley catheter is the preferred method for most clinicians. An indwelling catheter after catheterization is particularly important [13], because the urethral mucosa edema, bladder neck incarceration, and weak bladder contraction caused by the overstuffed bladder and overstretched detrusor muscle can make the patient unable to urinate after extubation. Our study shows that catheter indwelling for seven days can fully drain urine and improve clinical efficiency. With reasonable clinical care, such as daily cleaning of the urethra, instructing the patient to drink more water, and body position changes (knee-chest position; Figure 2), not only does it not increase the chance of urinary tract infection, but also it can relieve the pressure of the retroflexed uterus on the bladder and subside the urethral mucosal edema. Manual reduction during vaginal examination is another resetting method used as a first-line treatment. Use two fingers to gently press on the retroverted uterus at the posterior vaginal fornix and support the rectum with one finger while gently pressing on the suprapubic area of ​​the abdomen [4,14]. Manual reduction is not recommended after 20 weeks of pregnancy as it increases the risk of preterm birth [14]. If the above processing methods fail, invasive surgery, including colonoscopy-assisted repositioning and laparotomy or laparoscopy, may be considered. For a non-incarcerated uterus, it sticks out of the abdomen as pregnancy increases, which naturally relieves pressure on the bladder. For such patients, invasive procedures should be used with caution [11].

## Conclusions

AUR is a rare emergency that can occur at any time in pregnancy. Clinicians need to identify and diagnose in a timely manner based on symptoms and signs and combine them with images to quickly find the cause. Early individualized treatments can relieve bladder pressure in time, reset the uterus, and prevent spontaneous abortion and other destructive consequences for the mother and fetus. Close follow-up and patient education are also important for the recurrence of UR. These include limiting fluid intake before bed, avoiding the Valsalva maneuver, turning to a prone position, leaning forward when urinating, seeking immediate medical advice at the earliest warning sign, and more. 
